# Identifying potential three key targets gene for septic shock in children using bioinformatics and machine learning methods

**DOI:** 10.3389/fimmu.2025.1586584

**Published:** 2025-06-17

**Authors:** Wei Guo, Hao Chen, Feng Wang, Yingjiao Chi, Wei Zhang, Shan Wang, Kezhu Chen, Hong Chen

**Affiliations:** ^1^ Department of Pediatrics, First Affiliated Hospital, Heilongjiang University of Chinese Medicine, Harbin, China; ^2^ Department of Surgery, Heilongjiang Academy of Traditional Chinese Medicine, Harbin, China; ^3^ Department of Pediatrics, Harbin First Hospital, Harbin, China; ^4^ Ning ‘an Hospital of Traditional Chinese Medicine Pediatrics, Ning ‘an, China; ^5^ Graduate School, Heilongjiang University of Chinese Medicine, Harbin, China

**Keywords:** septic shock, children, potential gene, inflammation, machine learning

## Abstract

**Background:**

Septic shock in children is an infectious disease caused by low immunity, and its mortality is very high. Early prediction of the risk of death in children with septic shock is helpful for clinicians to judge the severity of the disease, take active treatment measures, and improve the adverse outcomes of patients. However, the mechanism of death from sepsis in children remains unclear. This study aims to use bioinformatics and machine learning algorithms to identify key genes and pathways associated with fatal sepsis in children, and provide theoretical basis for rational drug use in follow-up TCM treatment.

**Methods:**

Gene expression profiles were obtained from the GEO database (GSE4607) for 15 blank patients and 14 children with sepsis death. Differentially expressed genes (DEGs) were enriched by GO and KEGG pathways. Construct and visualize protein-protein interaction (PPI) networks to identify candidate genes responsible for fatal sepsis in children. Three kinds of machine learning models were established, and the candidate genes were screened by intersection to obtain the core genes with diagnostic value. ROC curve was drawn for core genes to clarify the diagnostic value of genetic markers.

**Results:**

Analysis of differences in the preprocessed dataset identified 83 genes, including 78 up-regulated genes and 5 down-regulated genes. 17 candidate genes were screened by protein interaction network analysis. Three machine learning algorithms LASSO, random forest (RF), and support vector machine recursive feature elimination (SVM-RFE) were used to finally screen out three core genes: CD163, MCEMP1 and RETN. CD163, MCEMP1 and RETN may jointly regulate complement and coagulation cascades, toll like receptor signaling pathway, graft versus host disease, type I diabetes mellitus.

**Conclusion:**

In this study, three core genes (CD163, MCEMP1 and RETN) that lead to sepsis death in children were screened out, providing a new understanding of the lethal mechanism of sepsis in children and a promising new therapeutic approach.

## 1 Introduction

Septic shock refers to a life-threatening condition of organ dysfunction. It is triggered by the body’s abnormal response to an infection, leading to severe circulatory, cellular and metabolic dysfunction, and is the most serious complication with rapid progression and high mortality (1, 2). It is estimated that there were 1.1 million sepsis related deaths worldwide, with a mortality rate of 148.1/100,000, accounting for 19.7% of the total number of deaths in that year, among which the mortality rate of children was higher than that of adults (3). Childhood has the highest lifetime incidence of sepsis, with an estimated 3 million deaths per year worldwide between 1990 and 2017 (4). The mortality rate in children with septic shock is between 17% and 32%. In developed countries, the case fatality rate of septic shock in children can reach 10% to 13%, in restricted areas, it can reach 18% to 24%, and in a few countries, it can even reach 34% to 58% (5). Due to the severe condition and high fatality rate of septic shock, reducing the fatality rate of septic shock has been the focus of the research of pediatric intensive care medicine in the world. Septic shock often leads to rapid deterioration in most children, and half of the fatalities take place within 48 hours after the onset (6). These observations suggest the need for early intervention before onset to reverse septic shock in children (7). In addition, survivors often suffer severe sequelae that affect long-term health-related quality of life. Therefore, the need to further investigate the key expression of septic shock and the importance of identifying potential biomarkers are becoming more and more obvious.

In the past few years, bioinformatics has emerged as a potent instrument for gaining a comprehensive understanding of the molecular mechanisms underlying diseases, as well as for pinpointing potential biomarkers and therapeutic targets. By comparing gene expression differences, transcriptional disparities and specific molecular pathways can be characterized. Gene expression analysis not only plays a crucial part in the molecular diagnosis of a wide range of human diseases but also demonstrates remarkable potential in the analysis of septic shock (8, 9). Machine learning (ML), through processes such as model training, optimization, and evaluation, has the ability to predict and identify unknown data. It offers valuable decision-making support for the research of disease pathogenesis and the formulation of preventive measures. As a result, it has revealed significant potential within the realm of bioinformatics. Studies have shown that ML can predict disease using related genes. However, few have identified the key gene associated with septic shock. Numerous studies have integrated bioinformatics analysis and machine learning algorithms to pinpoint disease - related genes that could potentially be linked to prognosis. Liang et al. used integrated bioinformatics and machine learning methods to elucidate 12 key genes related to sepsis and purine metabolism (10). In addition, Lin et al. suggested that DNMT1, TP53, and TLR8 might be considered as highly valuable biomarkers for the purpose of early diagnosis (11). The discovery of these key genes that contribute to the development of septic shock could be critical to understanding the factors and mechanisms that affect the disease. This will provide a basis for future research into the lethality of septic shock, ultimately reducing the potential risk of disease.

In the present study, we methodically employed bioinformatics tools and machine - learning methods based on a large sample of data to uncover septic shock-related expression profile data and an ML algorithm to identify a key septic shock-related gene. Download a microarray dataset of septic shock from the Gene Expression Omnibus (GEO). Subsequently, differential expression genes (DEGs) were analyzed in septic shock patients and control groups. The infectious shock hub genes screened from the differential genes were then analyzed by machine learning. The cross-gene and retrograde function and pathway were analyzed, and the relationship between proteins was studied. Finally, the key genes CD163, MCEMP1, and RETN were discovered in septic shock, which is expected to provide new insights into the pathogenesis of septic shock.

## 2 Materials and method

### 2.1 Screening and acquisition of original data

We searched for data in NCBI’s GEO database with the keyword “septic shock” and excluded chip data sets with sample size <6 using the species “Homo sapiens” as a screening criterion. The final data included in the analysis is GSE4607 and the platform number is GPL570. All cases included in the data were children under 10 years old. Expression data and related annotation files were collected for this group of data. We used 15 blank data and 14 fatal shock data in this dataset for systematic analysis. These datasets exhibit excellent data quality control, featuring complete matrices and comprehensive clinical information. The probes of these data are publicly accessible, and they also possess matrix information that can be effectively normalized. For the RNA-seq data, standard gene expression normalization and log2 conversion were carried out.

### 2.2 Screening for differentially expressed genes

The “Limma” package in R language was used to analyze the differential expression gene (deg) in the sample data of the blank group and the lethal shock group (12). A p value <0.05 or p value itself equal to 0.05 was considered statistically significant. In order to determine whether there was differential expression of genes, we adopted LogFC (log fold change) > 1 and adjusted the p0.05 standard. The ‘pheatmap’ and ‘ggplot2’ software packages were used to generate volcano maps and heat maps of differentially expressed genes (DEGs).

### 2.3 Data set path analysis

We analyze the pathway of differentially expressed genes analyzed from the blank group and septic shock group in the GSE4607 dataset. Use R language to draw the top 5 path maps for ascending and descending rankings, as well as path mountain maps.

### 2.4 GO and KEGG enrichment analysis

To explore the biological mechanism of the genes related to lethal septic shock, GO and KEGG enrichment analyses were conducted on all the selected differential genes (13). Subsequently, functional associations were established to gain a better understanding of the role played by the hub genes. It should be noted that items with P values less than 0.05 were regarded as significantly enriched. The final results were presented in the forms of bubble maps and heat maps.

### 2.5 Differential gene protein interaction network analysis and key core gene screening

The analysis of Protein-protein interaction (PPI) among Differentially Expressed Genes (DEGs) is grounded in the STRING database (https://cn.string-db.org/) (14). This database enables the search for relationships between proteins of interest. These relationships can include direct binding interactions or the co-existence within upstream and downstream regulatory pathways, facilitating the construction of a PPI network with intricate regulatory connections. The minimum required interaction score parameter is set at 0.04. Subsequently, the PPI network is visualized using Cytoscape (http://www.cytoscape.org).

### 2.6 Selection biomarkers by machine learning methods

In order to more accurately identify the characteristics that can be used as biomarkers for the diagnosis of infection shock, after preliminary screening of differentially expressed genes, we resorted to three machine learning algorithms: LASSO, random forest (RF), and support vector machine recursive feature elimination (SVM-RFE) (15, 16). These were employed to identify biomarkers originating from DEGs related to the diagnosis of septic shock. Specifically, the LASSO algorithm was implemented using the “glmnet” package, with the response type configured as a binomial and the regularization parameter (α) set to 1. In order to optimize the penalty parameter, cross-validation was performed tenfold in model training. The latter is carried out using the R software package “SVM-RFE”, with penalty parameter adjustment determined by 10x cross validation and minimum classification error. The default parameters were used in this script (cost = 10, cachesize = 500, scale = false, type = “C-classification,” kernel = “linear”). The Random Forest model was constructed using the ‘RandomForest’ (Version 4.7–1.1) package in R with 10-replicated tenfold cross-validation. As a randomization-based algorithm, it aims to avoid overfitting of a single decision tree and improves the overall model performance by utilizing multiple related decision trees generated from the same training set. In the context of this study, the genes selected as features were the top 30 genes.

### 2.7 Enrichment analysis of key gene sets

Gene Set Enrichment Analysis (GSEA) is carried out for the genes under consideration. The objective is to uncover the biological importance of the genes that can distinguish between different conditions or groups. In order to obtain a standardized enrichment score for each analysis, 1,000 different genome arrangements must be completed. To assess which KEGG pathways are significant, it is assumed that FDR <0.05 indicates substantial enrichment.

## 3 Results

### 3.1 Analysis of differential expression

**Figure 1:**
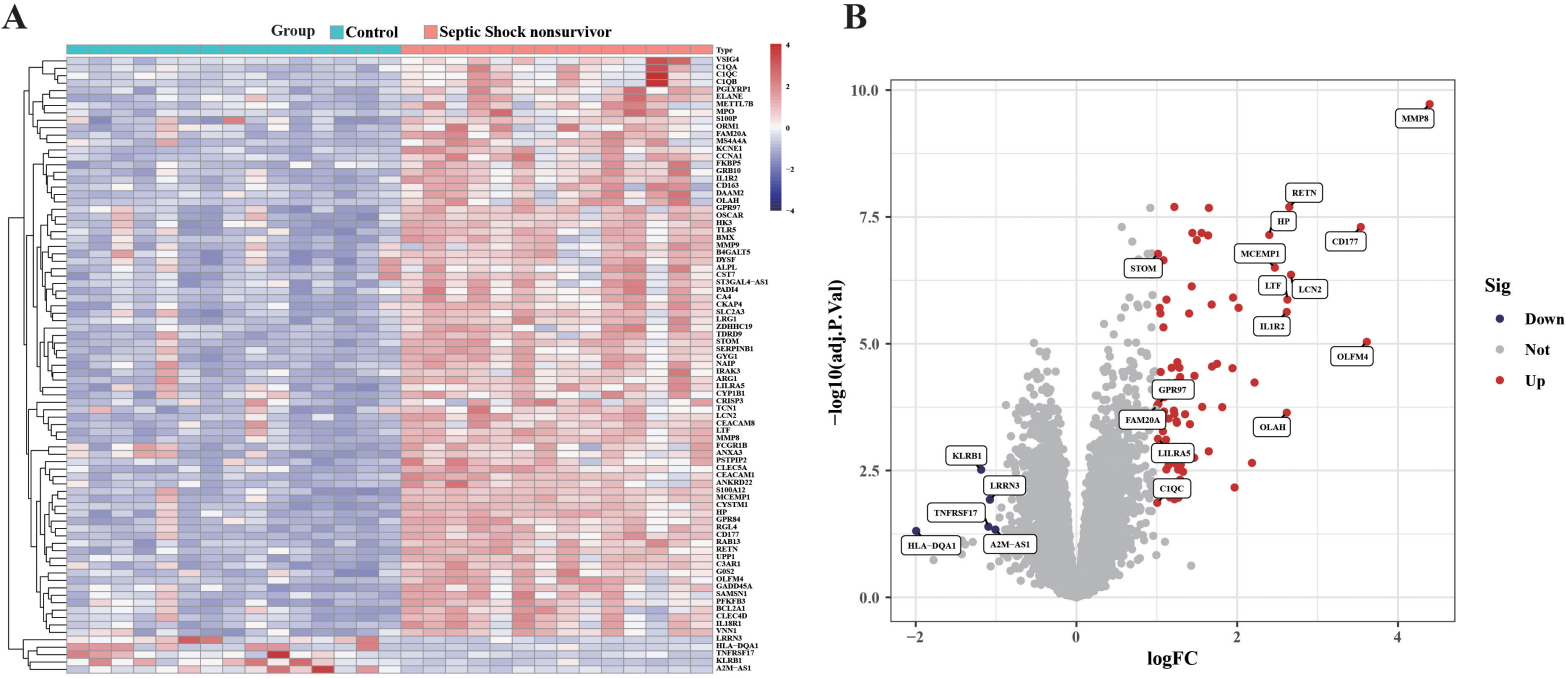
Differential gene study between blank group and sepsis death group in GSE4607 dataset. **(A)** Heatmap displaying the top fifty DEGs. **(B)** Volcano plots of DEG distributions.

A large amount of RNA-seq data from sample GSE4607 was analyzed with limma and DESeq2 R package for septic shock and normal tissue differential expression gene analysis. There were 78 up-regulated genes and 5 down-regulated genes in GSE4607 (adjusted p value < 0.05 & |logFC| | 1) (**Figures 1A, B**).

### 3.2 Data set path analysis

**Figure 2:**
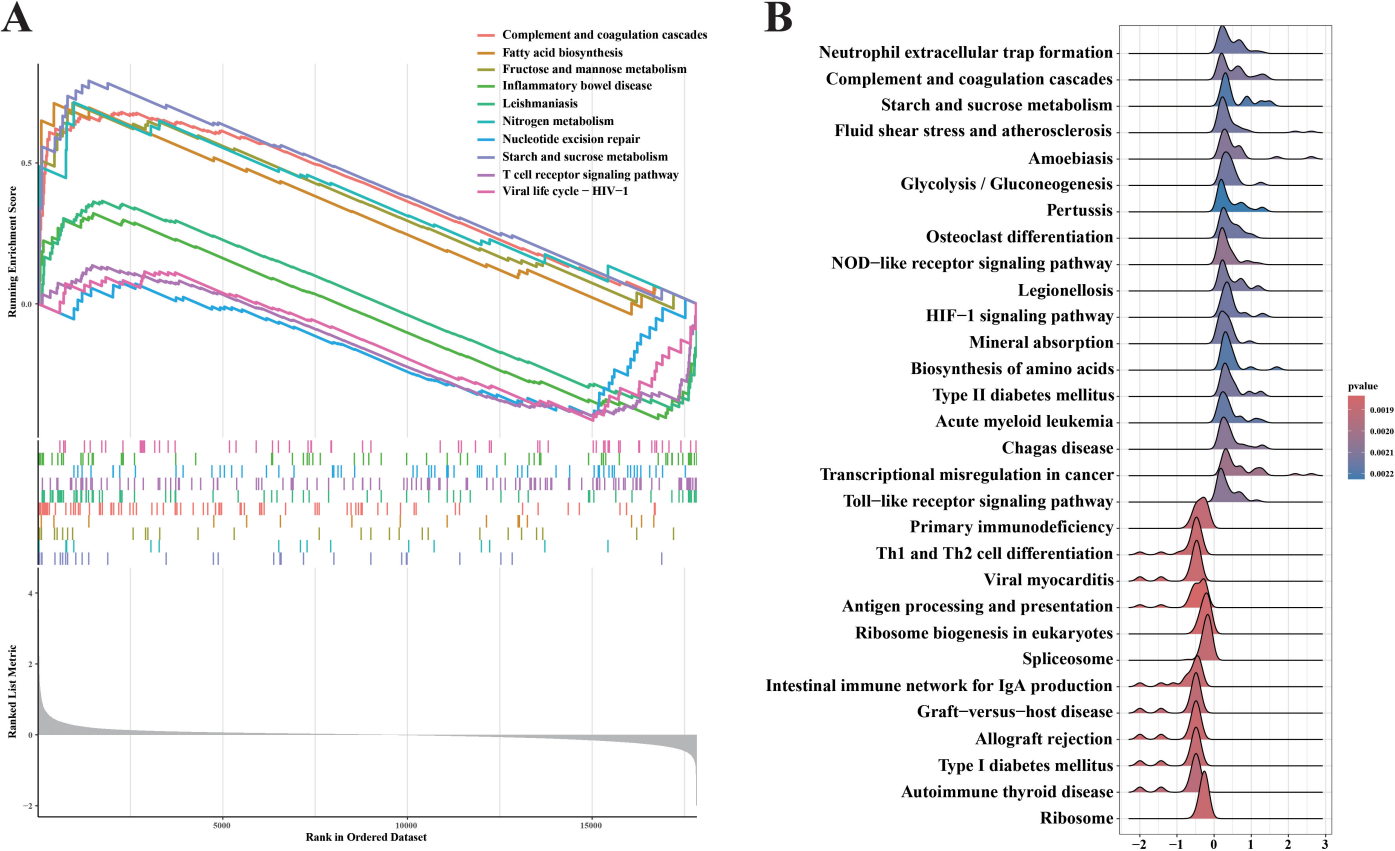
GSEA analyses result of the data set GSE4607. **(A)** Upregulated and Downregulated KEGG signaling pathways of the top 10 routes. **(B)** A GSEA investigation of each and every pathway.

Gene Set Enrichment Analysis (GSEA) is able to reveal the hidden biological pathways of complex diseases. We performed GSEA analysis on genes in the GSE4607 dataset, expecting to show a key pathway of significant enrichment in the septic shock context. **Figure 2** shows the top 10 pathways that show notable enrichment in genes in the GSE4607 dataset. To provide a detailed and comprehensive overview, we use KEGG analysis, a reliable resource for classifying and annotating biological pathways based on related gene and molecular interaction systems. **Figure 2A** shows the most abundant pathways among the genes identified in our study. It is worth noting that complement and coagulation cascades, Fatty acid biosynthesis, Fructose and mannose metabolism, Nitrogen metabolism, Starch and sucrose metabolism showed significant upregulation. These findings reveal the fundamental biological processes and molecular mechanisms that drive the occurrence and development of septic shock. Our GSEA also reveals interesting insights into the down-regulated pathway associated with septic shock. Inflammatory bowel disease, Leishmaniasis, Nucleotide excision repair, T cell receptor signaling pathway, and Viral life are shown the cycle-HIV−1 pathways, which show significant downregulation. To provide a comprehensive breakdown of gene enrichment pathways, we found that 18 pathways were positively correlated with gene expression and 12 pathways were negatively correlated with gene expression (**Figure 2B**). This visual depiction enabled us to obtain crucial insights into the particular genes underpinning each enrichment pathway. It unveiled the intricate network of molecular interactions responsible for the dysregulation witnessed in septic shock. These results offer additional clues regarding the dysregulated molecular cascade and abnormal cellular processes that play a role in the development of septic shock.

### 3.3 Differential gene enrichment pathway analysis

**Figure 3:**
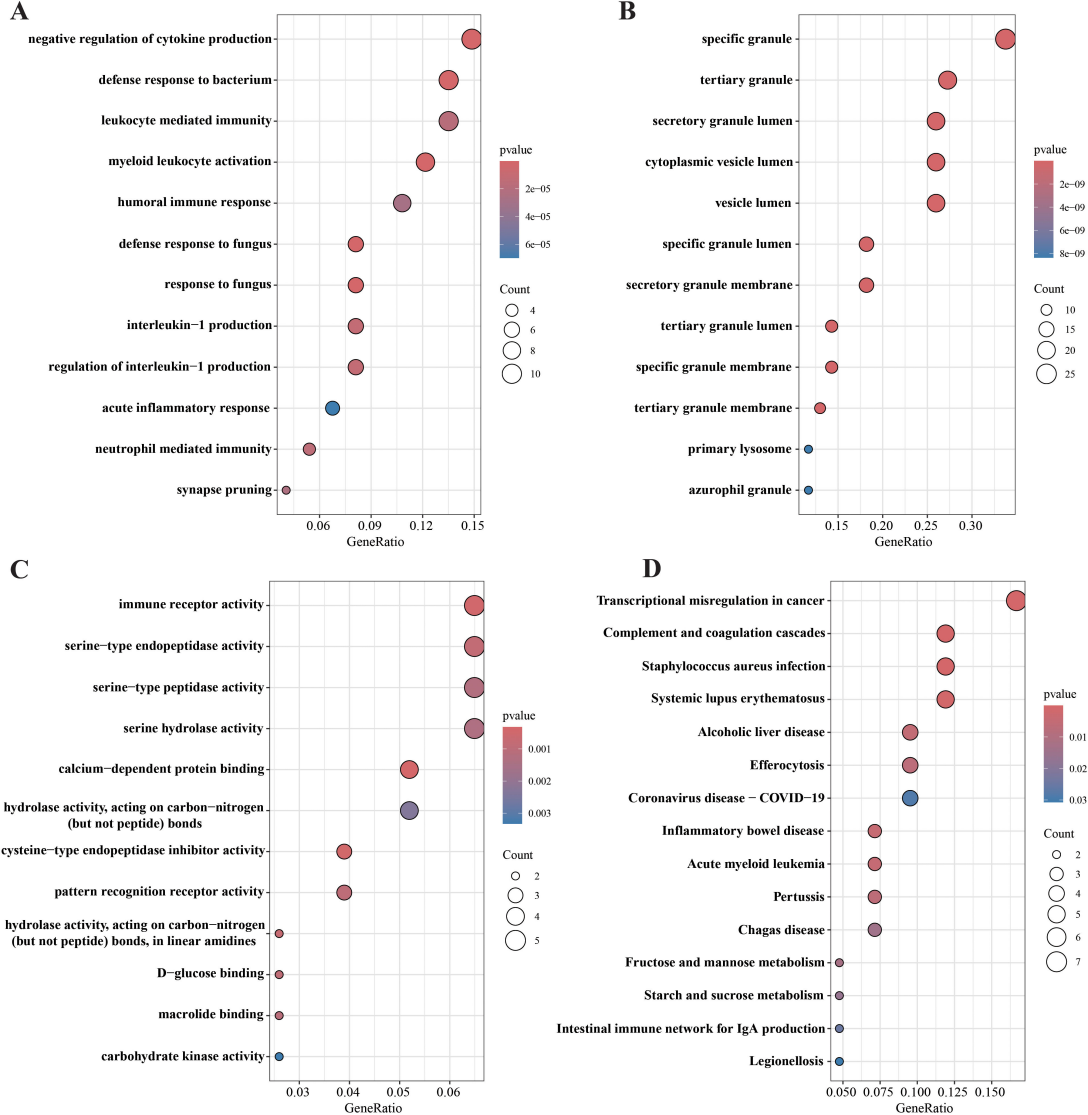
GO and KEGG pathway enrichment analysis of 83 DGEs. **(A)** biological processes (BP) analysis; **(B)** cellular components **(CC)** analysis; **(C)** molecular functions (MF) analysis; **(D)** KEGG enrichment analysis.

83 differential genes were subjected to Gene Ontology (GO) enrichment analysis, including BP, MF, and CC. First, biological processes (BP) of the screened genes were analyzed (**Figure 3A**). BP includes negative regulation of cytokine production, defense response to bacterium, leukocyte mediated immunity, myeloid leukocyte activation, humoral immune response, defense response to fungus, response to fungus, interleukin−1 production, regulation of interleukin−1 production, acute inflammatory response, neutrophil mediated immunity, synapse pruning. With that, We performed cellular components (CC) analysis on the screened genes (**Figure 3B**). CCs includes specific granule, tertiary granule, secretory granule lumen, cytoplasmic vesicle lumen, vesicle lumen, and specific granule lumen, secretory granule membrane, tertiary granule lumen, specific granule membrane, tertiary granule membrane, primary lysosome, azurophil granule. These molecular locations highlight the complex interplay between cellular structure and dysregulated gene expression patterns observed in septic shock, providing valuable insights into the underlying mechanisms. Next, we focus on molecular functions of differential gene enrichment (MFs) (**Figure 3C**). MF Includes immune receptor activity, serine−type endopeptidase activity, serine−type peptidase activity, and serine hydrolase activity, calcium−dependent protein binding, hydrolase activity, acting on carbon−nitrogen (but not peptide) bonds, cysteine−type endopeptidase inhibitor activity, pattern recognition receptor activity, hydrolase activity, acting on carbon−nitrogen (but not peptide) bonds, in linear amidines, D−glucose binding, macrolide binding and carbohydrate kinase activity.

In addition, using KEGG pathway analysis for 83 hypothetical targets, we generate a bubble map (**Figure 3D**) of the top 15 most abundant KEGG signaling pathways in descending order of FDR values to briefly illustrate the interaction of these KEGG signaling pathways and highlight their significance in the onset and progression of septic shock. These targets are significantly enriched in pathways associated with pathogenic infectious diseases, immune system diseases and intestinal inflammatory diseases.

### 3.4 PPI network analysis and identification of hub genes

**Figure 4:**
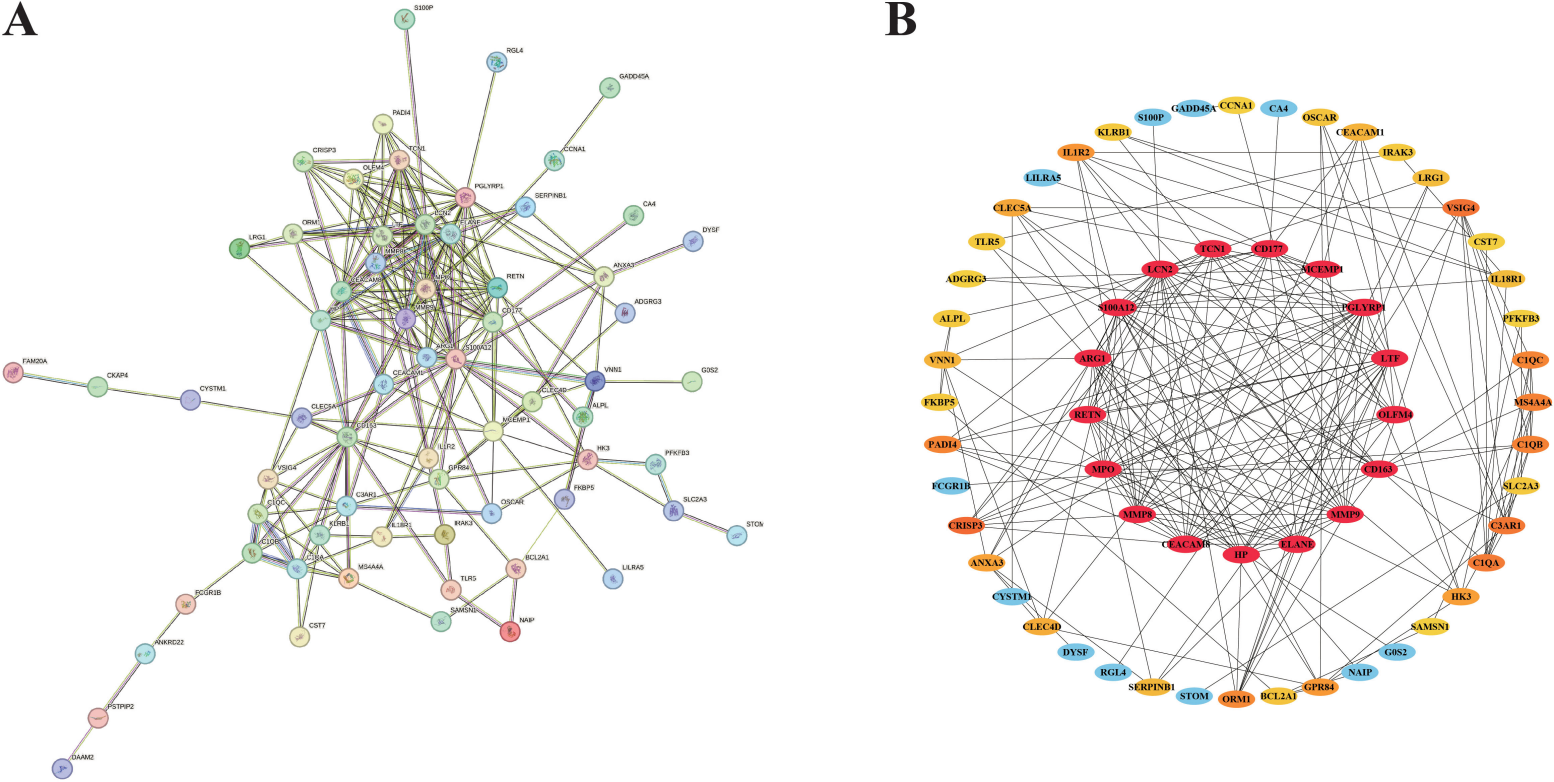
Results of PPI network analysis and identification of candidate nodes. **(A)** PPI network associated with DEGs. **(B)** Filter the candidate gene by PPI network.

Taking full advantage of the potential of the STRING website, we carefully created a more comprehensive protein-protein interaction (PPI) network (**Figure 4A**). The network diagram consists of 66 nodes and 223 edges. Subsequently, this network is imported into the Cytoscape application. With the utilization of the cytohubba plug-in, we can gain a deeper understanding of the topological characteristics of these nodes. This process enables us to generate an optimized and highly intuitive Protein - Protein Interaction (PPI) network diagram, as depicted in **Figure 4B**. This visualization not only clearly demonstrates the complex interactions among the potential targets identified in our research but also effectively conveys the significance of each node visually through color-coding. Through the analysis, 17 key genes related to septic shock were identified.

### 3.5 Screen key gene results based on three kinds of machine learning

**Figure 5:**
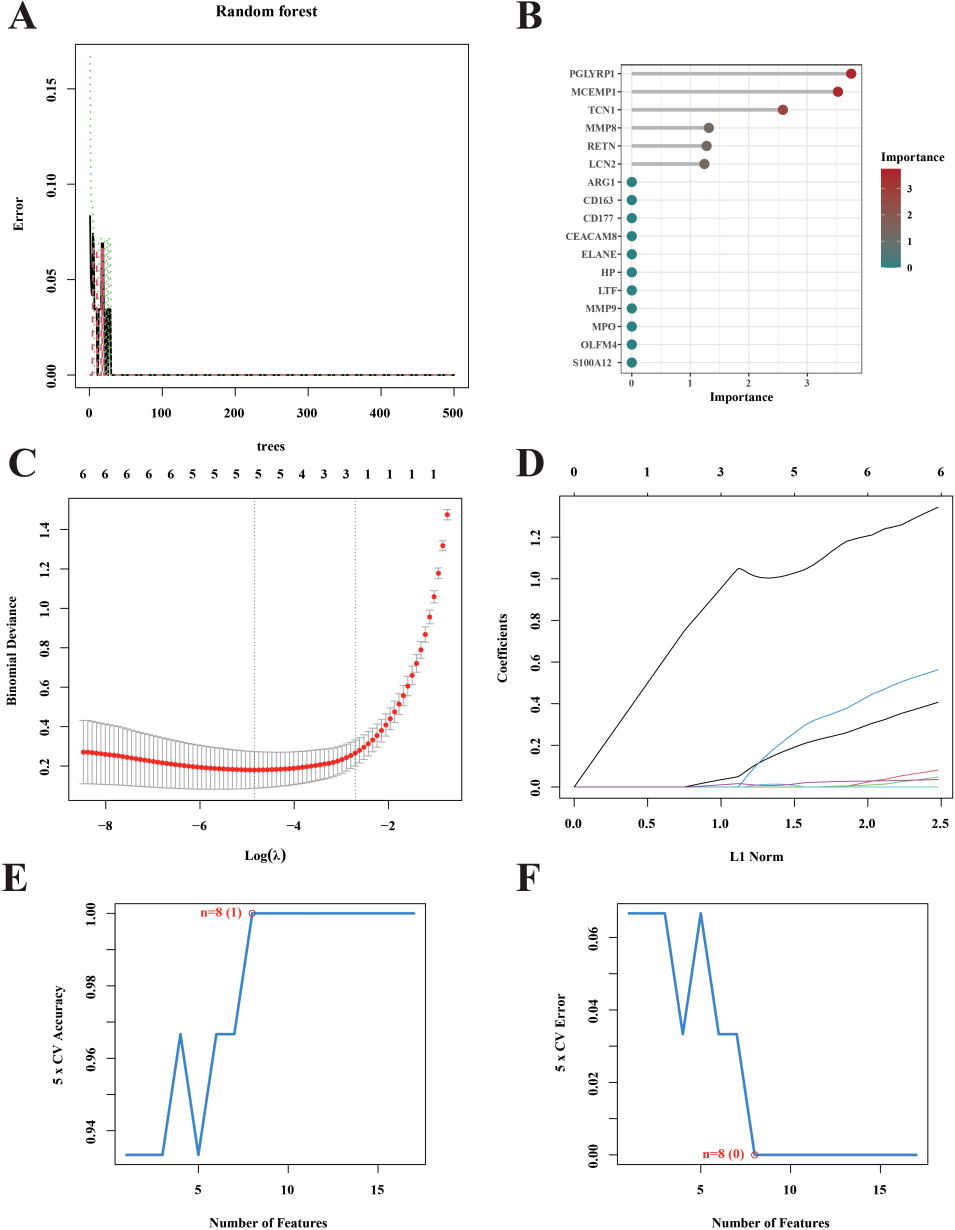
Screening for hub septic shock. **(A)** The genes were selected and ranked according to the importance scores assigned by the random forest algorithm in the context of septic shock. **(B)** The Gini coefficient method random forest classifier was used to filter results. **(C)** Tenfold cross-validation was performed to identify the optimal tuning parameter (λ). **(D)** LASSO coefficient profiles of the 5 genes. **(E, F)** Curves portraying the error rate and accuracy rate of variable selection.

We used three machine learning methods (lasso model, SVM model and RF model) to further screen and validate 17 key genes. According to the random forest algorithm, a total of 10 feature genes PGLYRP1, MCEMP1, TCN1, MMP8, RETN, LCN2, ARG1, CD163, CD177 and CEACAM8 were screened out (**Figures 5A, B**). The findings obtained from the Lasso model show that the existence of septic shock is related to the expression of five genes, including CD163, MCEMP1, MPO, RETN, and S100A12 (**Figures 5C, D**). During our research, to generate feature vectors from the data we’d amassed, we made use of a powerful machine - learning method, namely a Support Vector Machine (SVM). These feature vectors represent the genetic feature and pattern of septic shock. By means of this intricate process of data filtering and analysis, we managed to unearth eight of the most crucial genes closely related to septic shock (LTF, MMP8, CEACAM8, MCEMP1, HP, RETN, CD163, and PGLYRP1) (**Figures 5E, F**).

### 3.6 Identification of characteristic genes

**Figure 6:**
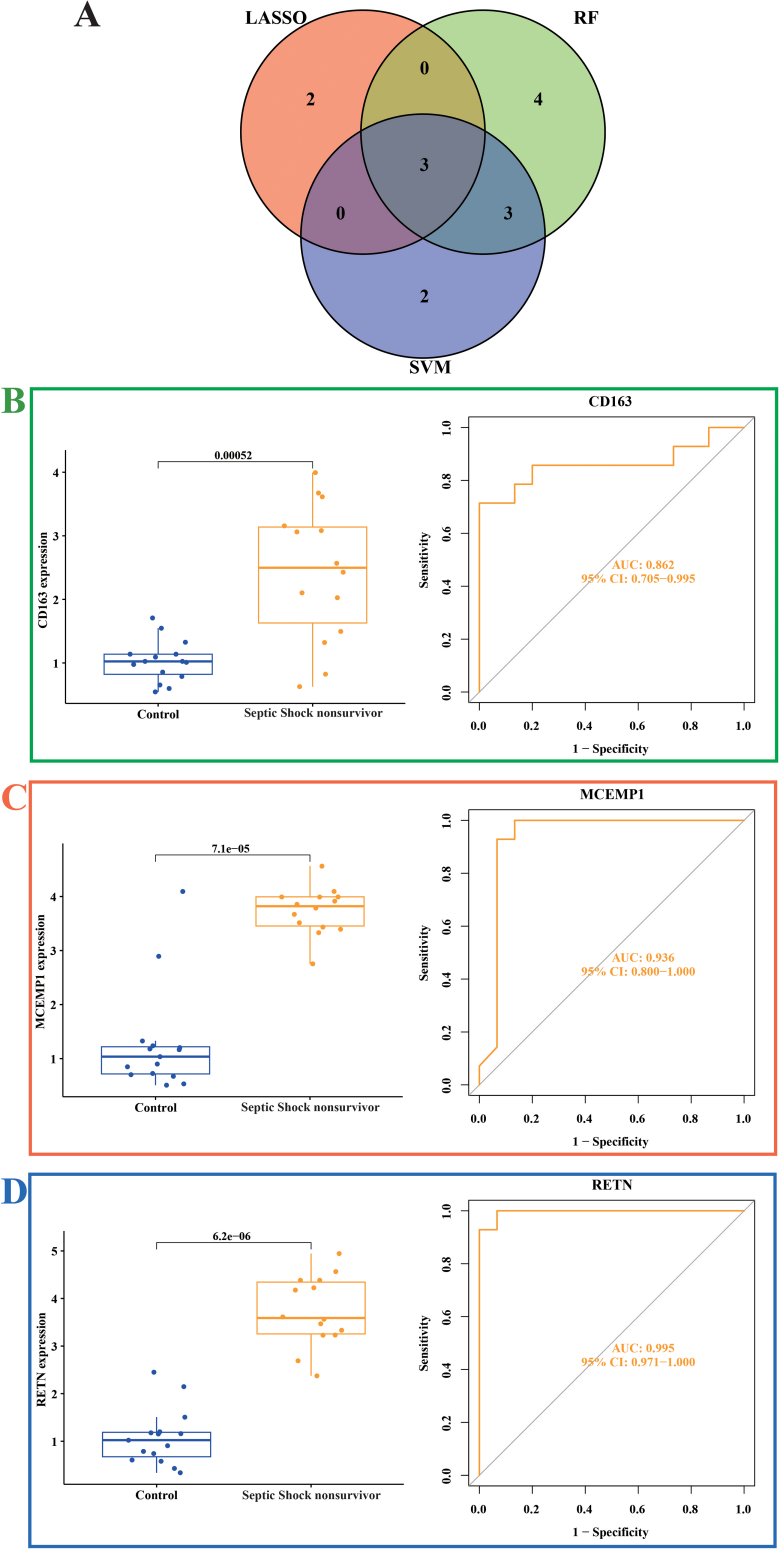
Expression of related biomarkers of septic shock **(A)** Venn diagram of the overlapping hub genes between the random forest, LASSO algorithm and SVM-RFE algorithms in septic shock. **(B)** Expression of the CD163 gene signature and ROC curves of classification models in the GSE4607 dataset. **(C)** Expression of the MCEMP1 gene signature and ROC curves of classification models in the GSE4607 dataset. **(D)** Expression of the RETN gene signature and ROC curves of classification models in the GSE4607 dataset.

We selected the genes outputted by three diverse machine learning models and subsequently carried out an intersection analysis on them. Therefore, we focused our research on three genes, CD163, MCEMP1 and RETN (**Figure 6A**). Compared with the control group, the expressions of CD163, MCEMP1, and RETN genes were significantly higher in the septic shock group. We evaluated the diagnostic efficacy and model accuracy of each gene using the AUC values of the internal dataset. This method of analysis enabled us to assess, in a quantitative manner, the predictive capabilities of CD163, MCEMP1, and RETN, and to determine their ability to distinguish septic shock patients from healthy individuals. In the GSE4607 dataset, the AUC value of the CD163 classification model is 0.862 (**Figure 6B**), that of the MCEMP1 classification model is 0.936 (**Figure 6C**), and that of the RETN classification model is 0.995 (**Figure 6D**). The results suggest that CD163, MCEMP1, and RETN has the possibility of acting as a trustworthy diagnostic marker, which can offer valuable insights into the existence and development of septic shock.

### 3.7 Functional enrichment analysis of CD163, MCEMP1 and RETN

**Figure 7:**
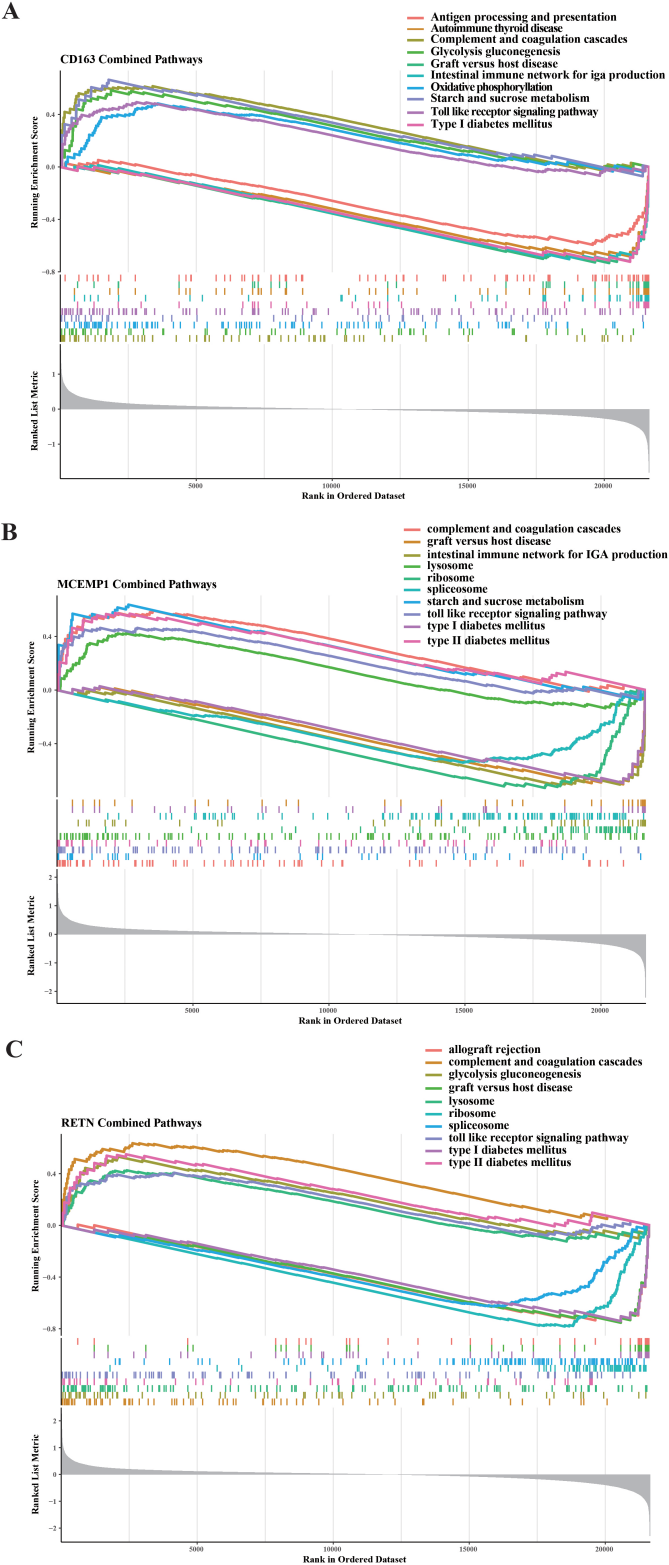
GSEA was carried out on the selected feature genes. Regarding the KEGG pathway analysis, the top five upregulated and downregulated pathways were determined for **(A)** CD163, **(B)** MCEMP1, and **(C)** RETN.

To uncover the functional implications and potential molecular pathways associated with CD163, MCEMP1, and RETN, we performed a rigorous GSEA analysis. We observed that CD163 was mainly involved in upregulating pathways including complement and coagulation cascades, glycolysis gluconeogenesis, starch and sucrose metabolism, and toll like receptor signaling pathway and oxidative phosphorylation. We also found that the down-regulated pathways affected by CD163 included antigen processing and presentation, autoimmune thyroid disease, graft versus host disease, and intestinal pathways immune network for IGA production and type I diabetes mellitus (**Figure 7A**). We observed that MCEMP1 is mainly involved in the up-regulation pathway, including complement and coagulation cascades, lysosome, starch and sucrose metabolism, toll like receptor signaling pathway, type II diabetes mellitus. We also found that the down-regulated pathways affected by MCEMP1 included graft versus host disease, intestinal immune network for IGA production, ribosome, spliceosome, and type I diabetes mellitus (**Figure 7B**). We observed that RETN is mainly involved in the up-regulation pathway, include complement and coagulation cascades, glycolysis gluconeogenesis, lysosome, toll like receptor signaling pathway, and type II diabetes mellitus. We also found that down-regulated pathways affected by RETN include allograft rejection, graft versus host disease, ribosome, spliceosome, type I diabetes mellitus (**Figure 7C**). CD163, MCEMP1 and RETN may co-up-regulate complement and coagulation cascades and toll like receptor signaling pathway. graft versus host disease and type I diabetes mellitus were down-regulated. These findings highlight the global role of CD163, MCEMP1, and RETN in these molecular pathways and provide a plausible explanation for their functional significance in septic shock.

## 4 Discussion

Septic shock is accompanied by an immune response to the pathogen, manifested by activation of pro-inflammatory and anti-inflammatory mediators (17). Dysregulation of the immune system is one of the main reasons for the high mortality (18). As a clinically diagnosed critical disease, septic shock has three important factors associated with death: first, early identification of patients with suspected septic shock; second, immediate and active treatment of patients with septic shock; third, attention should be paid to clinical indicators, laboratory indicators and hemodynamic monitoring during treatment (19). These factors are closely related to doctors’ cognition of the diagnosis and treatment strategy of septic shock in children and the allocation of pediatric intensive care resources. Therefore, the use of bioinformatics and machine learning methods to dig deeper into the key indicator proteins of childhood sepsis, and establish the possibility of their potential treatment options, to make accurate diagnosis of the disease in the first time, and create favorable conditions for the development of effective treatment. In this study, we used mRMR and LASSO logistic regression to screen and validate three key genes, namely CD163, MCEMP1 and RETN.

CD163 is a cell membrane surface molecule with an anti-inflammatory phenotype that is specifically expressed by macrophages and is involved in innate immunity as a receptor for the hemoglobin-contactoglobin (Hb-Hp) complex (20, 21). It exists in two distinct forms: the soluble form, sCD163, which is present in serum or plasma, and the membrane-associated form, mCD163. The macrophage-associated CD163 receptor plays a crucial role in the uptake and endocytosis of the hemoglobin (Hb) or the hemoglobin-haptoglobin (HB-HP) complex. The expression of the sCD163 receptor is regulated by tumor necrosis factor-α (TNF-α) and interleukin-10(IL-10). Enzyme-linked immunosorbent assay (ELISA) was used to detect serum of sepsis patients admitted to ICU on different days (1 day, 3 days, 5 days), and the results showed that septic shock was associated with the highest concentration of CD163. The admission value of CD163 has a significant impact on the prediction of mortality in sepsis patients (22). In addition, Dan et al. used CD163 as an important functional marker in their studies on the distribution and inflammatory phenotype of circulating monocyte subsets in patients with sepsis (23). In Kjærgaard’s study, the expression of CD163 monocytes in non-surviving patients with septic shock was higher than that of surviving patients at ICU admission and surviving patients during observation (24). Existing studies confirm that CD163 plays a critical role in the development and development of septic shock in adults. Based on an in-depth analysis of pediatric sepsis cohort data, this study is the first to identify a potential regulatory role the CD163 gene in pediatric septic shock progression. These findings suggest that the CD163 gene plays an important role in the pathogenesis of septic shock in different age groups, providing new scientific evidence for the study of mechanisms and targeted interventions across age groups of the disease.

Mast cell expressed membrane protein 1 (MCEMP1) is responsible for the encoding of single-pass transmembrane protein and is involved in the regulation of MC differentiation activity or immune response (25). MCs worsens septic disease by disrupting phagocytic cell activity of resident macrophages, thereby enabling the proliferative activity of local and systemic microbial factors. Chen et al. confirmed that Mir-125-mediated inhibition of MCEMP1 can reduce the levels of serum TNF-a, IL-1b and IL-6, and programmed cell death promotes the activity of T white blood cells, thereby reducing the immune activity of septicemia mice (26). In previous studies, we observed that MCEMP1 was elevated in a mouse sepsis model and a human LPS-treated macrophage model (27). These findings suggest that MCEMP1 may be a potential diagnostic marker of septic shock. At the same time, blocking the overexpression of the MCEMP1 gene could potentially serve as a treatment alternative for patients suffering from severe sepsis or septic shock.

Circulating myeloid cells produce the cytokine resistin (RETN) is an adipose tissue-specific secretion factor encoded by the RETN gene. It is a cysteine-rich peptide hormone that is chiefly expressed within adipose tissue and assumes an essential role in a variety of physiological and pathological processes. RETN was initially identified in mouse adipose cells and has been associated with the development of diabetes. In human beings, macrophages and neutrophils are the primary producers of RETN (28). It is believed that its capacity to activate endothelial cells contributes to the pathogenesis of atherosclerosis (29). This process may be mediated by toll-like receptor signaling pathways. Elevated levels of proinflammatory adipokine RETN have been observed during ICU sepsis (30) and have been studied as a potential indicator of neonatal sepsis. It has been hypothesized that RETN is involved in the pathogenesis of sepsis through its promotion of neutrophil-endothelial cell adhesion. Prior studies have demonstrated that, *in vitro*, RETN stimulates the expression of white blood cell adhesion molecules, namely ICAM-1 and VCAM-1, in endothelial cells. In the *in vivo* environment, these receptors are cleaved and released into the bloodstream in a soluble form. This soluble form can serve as an indirect indicator of endothelial cell activation (31). However, the potential role of RETN as a mediator of endothelial cell activation in clinical cases of sepsis remains unexamined.

In terms of pathophysiological mechanism, septic shock belongs to distributed shock. Under the invasion of pathogen infection, inflammatory mediators such as histamine, interleukin, 5-hydroxyserotonin and superoxide free radicals are released in a waterfall level, such as blood, which is manifested as significantly increased capillary permeability in the body, and peripheral vascular dilatation and reduced resistance (32, 33). Most BP analyses in this study focused on inflammatory responses, bacterial and fungal defense responses. Usually, mannose binds to lectin-associated serine proteases to activate the lectin pathway of the complement system, which is an important component of the dysregulated immune response in sepsis (34). In our study, we found that the main participants were immune receptor activity and serinase activity. KEGG analysis results included complement and coagulation cascade, staphylococcus aureus infection, systemic lupus erythematosus, coronavirus disease-COVID-19, inflammatory bowel disease, acute myeloid leukemia, transcription dysregulation of intestinal immune network IgA and Legionnaires’ disease. All of these pathways are associated with septic shock. Taken together, these findings further highlight the complex interplay between sepsis and inflammatory responses and bacterial and fungal defenses, highlighting inflammatory pathways as potential therapeutic targets.

Our study still has limitations. First, the small sample size of the dataset may lead to limitations in data analysis and one-sided results. To address this problem, we actively collect data, but data on septic shock in children are scarce and most data are incomplete. Therefore, our results require further validation by other analytical methods. While large clinical cohort studies are required to assess the three key genes identified in this study, this study does demonstrate the potential of these new approaches. In addition, the case data used in this study were incompletely collected, including, but not limited to, the patients’ gender, ethnicity, geographic location or comorbidities. The lack of this information makes it impossible to classify samples in more detail, which can lead to potential bias in the results. We therefore believe that future studies should consider model training and validation for people from different gender, ethnic and geographical backgrounds. In addition, the complexity and interpretive challenges of the model may limit their reproducibility and practical application in this area.

## 5 Conclusion

Based on the dataset of septic shock in the public database, we used bioinformatics in combination with machine learning to explore the potential target for septic shock, and found that the proteins CD163, MCEMP1, and RETN are associated with the risk of septic shock. Compared with a healthy control group, septic shock patient had significantly higher expression levels of CD163, MCEMP1, and RETN. Patients with higher levels of CD163, MCEMP1, and RETN had higher mortality, and this lower survival rate was found to be associated with septic shock risk factors that were independent and specific. Using machine learning models to predict the potential mechanisms and targets of CD163, MCEMP1, and RETN proteins, CD163, MCEMP1 and RETN may jointly regulate complement and coagulation cascades, toll like receptor signaling pathway, graft versus host disease, type I diabetes mellitus. This research offers a novel perspective regarding the potential mechanism underlying septic shock. Additionally, it presents a fresh research lead for identifying the therapeutic target of septic shock.

## Data Availability

The original contributions presented in the study are included in the article. Further inquiries can be directed to the corresponding author.
